# Developing a tool for measuring the disaster resilience of healthcare rescuers: a modified Delphi study

**DOI:** 10.1186/s13049-020-0700-9

**Published:** 2020-01-20

**Authors:** Xiaorong MAO, Alice Yuen LOKE, Xiuying HU

**Affiliations:** 1 Institute for Disaster Management and Reconstruction, Sichuan University, Chengdu, Sichuan China; 2 Sichuan Academy of Medical Sciences & Sichuan Provincial People’s Hospital, Chengdu, Sichuan China; 3 School of Nursing, the Hong Kong Polytechnic University, Hung Hum Kowloon, Hong Kong; 4 School of Nursing / West China Hospital, Sichuan University, Chengdu, Sichuan China

**Keywords:** Disaster resilience, Healthcare rescuers, Modified Delphi technique

## Abstract

**Background:**

Disaster resilience is an essential personal characteristics of health rescue workers to respond to disasters in an effective manner, and maintain a state of adaptation after deployment. It is essential for disaster managers to recruit, assess, and prepare healthcare rescuers with this characteristic. A specific tool for measuring the disaster resilience of healthcare rescuers has yet to be devised.

**Objective:**

The purpose of this study was to establish the content validity of a tool for measuring the disaster resilience of disaster rescue workers.

**Methods:**

A modified Delphi approach was employed. Experts in disaster work and research were invited to rate the domains and items of a prototype tool for measuring disaster resilience in healthcare rescue workers. The panel of experts rated the relevance of the items using a 4-point Likert scale. The median and interquartile range, as well as the level of agreement, were calculated for each item using the Kendall coefficient W, to assess the consensus of the experts. The content validity index (CVI) was calculated to assess the content validity of this tool.

**Results:**

A total of 22 and 21 experts were involved in the first and second rounds of this modified Delphi study (response rate of 91.7 and 95.5%), respectively. After two rounds of expert query, an eight-domain and 27-item disaster resilience measuring tool was established. The median range of all of the included items was 3.50 to 4.00 and the interquartile range was 0.00 to 1.00, and all items achieved ≥85% agreement. The Kendall coordination coefficient W was 0.21 and 0.33 in the first and second rounds, respectively, with *P* < 0.01. The I-CVI ranged from 0.85 to 1.0, while the S-CVI/UA and S-CVI /Ave were 0.69 and 0.97, respectively.

**Conclusion:**

Consensus was reached on a disaster resilience measuring tool covering 27 items. The content validity of this tool for measuring the disaster resilience of healthcare rescuers was excellent. This tool is validated and ready to be tested in a pilot study to assess its psychometric properties.

## Background

Resilience is regarded as the ability to “bounce back” from disaster, sustaining well-being and life satisfaction without negative psychological symptoms over time [1]. Resilience is also considered one of protective factors against occupational burnout [2, 3]. It has been suggested that healthcare rescue workers who have a high level of disaster resilience are not only less likely to suffer from negative psychological problems such as anxiety and depression, and posttraumatic stress disorder (PTSD), but also work more effectively [4–6]. Thus, disaster resilience is essential for the health and well-being of both disaster rescuers and the survivors of a disaster. It is desirable for disaster rescue workers to be recruited from among those with a high level of resilience.

An annual average of 77,144 deaths due to disasters were recorded between 2000 and 2017 [7]. Recent data show that 10,373 lives were lost in 2018 because of catastrophic events such as earthquakes, tsunamis, and volcanic activity, which is a demonstrable decline. The prevalence of Post-traumatic stress disorder PTSD among healthcare disaster rescuers was reported to be as high as 28.6% at 8 months after the Yushu earthquake in China [8]. Nurses who responded to the 2008 Wenchuan earthquake were at higher risk of suffering from PTSD (30%), compared to other healthcare rescuers [9].

Studies have also suggested that factors protective of resilience, such as social support and coping strategies, can be modified, learned, or cultivated through intervention programs [10, 11]. Thus, it is possible to design and develop interventions to foster the resilience of rescuers who are at a high risk of suffering from negative psychological consequences. There is also a need to have a valid and reliable tool for measuring disaster resilience, for use in recruiting disaster rescue workers and in evaluating the effectiveness of interventions that have been developed to enhance the resilience of individuals.

Existing instruments, such as the Connor-Davidson Resilience Scale (CD-RISC) [12, 13] and the Resilience Scale [14] have been used in studies to measure resilience among disaster rescue workers. However, these instruments were originally developed based on the general population or on patients with psychological disorders rather than specifically on rescue workers. Instruments that are “borrowed” from other populations or contexts may not be appropriate for the specific population or context of interest [15]. It is thus inappropriate to use an existing non-specific measuring tool to screen rescue workers for resilience in the recruitment process, or to evaluate the effectiveness of intervention programs aimed at fostering resilience in disaster rescue workers. As there is no specific resilience scale that can serve as a “gold standard,” and no specific instrument to measure the disaster resilience of rescue workers, the use of “borrowed” instruments on resilience has led to confusion in disaster management and research. It is therefore imperative to develop a valid and reliable instrument specifically for assessing the disaster resilience of disaster rescue workers in the context of disaster deployment.

### Validation of a prototype tool

To our knowledge, there is no consensus on a framework for assessing disaster resilience in healthcare rescuers. A prototype tool for measuring the disaster resilience of rescue workers was developed by the research team. The tool was developed based on an extensive review of the literature on the characteristics of resilience among disaster rescue workers, a concept analysis of the concept “disaster resilience” and a focus group interview study of disaster healthcare rescuers, who were asked to give their views on disaster resilience [16]. Based on the results of these works, a scoping review of the tools for measuring the resilience of adults was conducted, and a prototype disaster resilience tool for healthcare rescuers was developed. The scale consists of eight domains: optimism, altruism, preparations for disaster, social support, perceived control, self-efficacy, coping strategies, and positive growth.

## Methods

This study adopted a modified Delphi method to validate the instrument. A modified Delphi was a kind of technique of establishing consensus among a panel of experts on a topic of interest [17]. A traditional Delphi process begins with an open-ended questionnaire, which is time-consuming and usually leads to a low response rate [18, 19]. In a modified Delphi study approach, experts are consulted in the very first round using a structured questionnaire developed based on extensive reviews of the literature and / or on a focus group interview study [20]. The use of a modified Delphi process is appropriate when basic information concerning the target issue / topic is available and usable [17].

An online modified Delphi approach [19, 21] was the approach adopted in this study to obtain the judgment of a panel of independent experts on this specific issue, on which there is insufficient knowledge and research evidence to provide guidance on practice [22].

The aim of this study was to refine the domains and items of the prototype tool for measuring disaster resilience among healthcare rescuers and to establish the content validity of the items in that tool.

### Panel selection

A purposive and criterion-based sampling method [23, 24] was adopted for selecting the members of the panel.

In a Delphi study, the experts represent those from various geographical locations [25], who are knowledgeable [19, 24, 26], possess professional and special expertise [27], have attained a certain level of educational status [28], and are willing to participate in the survey [26, 28, 29].

It has been suggested that a sample of experts can be identified through conferences [30] and published literature [31]. The potential experts for this study were acquaintances from international conferences / workshops on disaster nursing/management, such as the World Society of Disaster Nursing (WSDN, Germany, October 2018) and the Asia Pacific Emergency and Disaster Nursing Network (APEDNN, Cambodia, November 2018), as well as internationally known experts identified from published research studies / books on topics related to disaster healthcare.

The experts who were involved in academic and / or empirical work on disasters were selected in accordance with the purpose of this project [32]. They are from various geographical locations: the United States of America, the United Kingdom, Australia, Japan, South Korea, Taiwan, Hong Kong, and mainland China. The members of the panel of experts in the present study were selected from different countries based on the following criteria for inclusion: [1] the possession of a bachelor’s degree or above [2]; relevant experience / significant contributions in disaster management, disaster nursing / medicine / healthcare, or disaster-related research; and [3] at least 5 years of disaster-related clinical or academic experience. Those who could not read English, or could not be reached by electronic means via computer / email were excluded.

In the literature on Delphi studies, it is suggested that ten to fifteen subjects could be sufficient [17, 26]. It is also common for three experts to be considered sufficient for assessing the content validity of an instrument that has been developed [33]. Our aim was to recruit at least 15 international experts to take part in this study.

### Format of the prototype tool for validation

The experts were invited to provide comments on the domains / components of the tool for measuring the disaster resilience of healthcare rescuers by rating the relevance of each item of the prototype tool on a 4-point Likert scale, with 1 = not relevant, 2 = somewhat relevant, 3 = quite relevant, 4 = highly relevant [34]. Although a 3-or 5-point rating scale is the commonly adopted format for validation, a 4-point Likert scale was adopted to obtain an even number of possible responses to avoid a neutral and ambivalent result at mid-point [33].

A pilot test of the validation form of the prototype tool was conducted among three experts who were not included in the panel of expert in the Delphi rounds. The pilot was to estimate the time required to complete the form and to ensure the clarity of the items. The experts suggested that information on the background and aims of this study should be provided, and information the background and demographics of the experts in the Delphi survey should be collected. Some minor clarifications were made to the wording of the items.

### Data collection procedure

At the commencement of the Delphi expert query, an invitation letter, information sheet with an explanation of the background and aim of the Delphi survey, together with the prototype tool survey form, were sent to the experts via email. These experts were those whom the researchers had approached while attending various conferences or whom they had identified from the literature and contacted by email. All of them agreed to take part in this Delphi query.

The panelists were asked to rate the relevance of each item on a 4-point Likert scale. The experts were also given the opportunity to suggest additional domains and items that might not have been included in the tool, and to give comments on the tool at the end of the survey form. As it has been suggested that ten to fourteen days should be a sufficient interval between rounds of assessment for expert query [35], the experts were given 2 weeks to return their ratings and comments. An email of reminder was sent to those in the panel who had not given their feedback after 2 weeks. If there was still no response within the next 2 weeks (4 weeks in total), it was concluded that the expert was not available or no longer interested in taking part in the study, and no further attempts were made to contact that person.

There was a two-week interval between the rounds of the Delphi survey. During this period, the feedback from the experts was summarized, scrutinized, and studied to refine the prototype tool. This feedback was also sent to the panel of experts in the next round. Only those who took part in the first round were invited to take part in the subsequent round(s) of the Delphi survey. In those subsequent round(s), the panelists were asked to rate the items using the same criteria for assessment described earlier.

The number of rounds depended on the level of consensus reached [19, 36], and the amount of time available [24]. Recent evidence has shown that two to three rounds are sufficient in a modified Delphi study [37–40]. The number of rounds of surveys in this study ceased when a consensus was reached on all items, as indicated when 70% of the experts reached an agreement [41–43].

### Statistical analysis

After the completion of each round of the Delphi survey, the data were inputted for statistical analyses into SPSS (Statistical Package for the Social Scientists) software version 25.0 for Windows (IBM Corp., New York, NY, USA). Consensus is one of the most contentious components of the Delphi method [36]. The median and interquartile range, as well as the level of agreement, were calculated to evaluate the consensus for each item in this Delphi research [44]. A consensus was considered to have been reached on the inclusion of an item in the disaster resilience scale if the median of the item was up to 3.25 on a 4-point scale [17], the interquartile range was less than 1 [45, 46], and the level of agreement was at least 70% [19]. The Kendall coefficient W test was adopted to evaluate the consensus on agreement among the panel of experts [47]. A two-tailed *p*-value of < 0.05 was considered statistically significant.

The content validity was calculated using the content validity index (CVI) [33]. Content validity was computed for each item (I-CVI) as well as for the overall scale (S-CVI), including the universal agreement (S-CVI/UA) and average (S-CVI/Ave). In this study, both I-CVI and S-CVI were calculated, and the values of statistical significance for I-CVI, S-CVI/UA, and S-CVI /Ave were set at ≥0.78, 0.8, and 0.9, respectively [48].

### Ethical considerations

Ethical approval for this study was obtained from the Human Research Ethics Committee of the School of Nursing, the Hong Kong Polytechnic University (HSEARS20190102004), and the West China Hospital, Sichuan University (2019#65). The experts were informed that their participation in this Delphi study was voluntary. Experts who returned their ratings of the tool were considered to have given their implied consent to participate in this study. In our study, only the researchers knew the name of the experts, and no individuals are identified in the report.

## Results

**Fig. 1:**
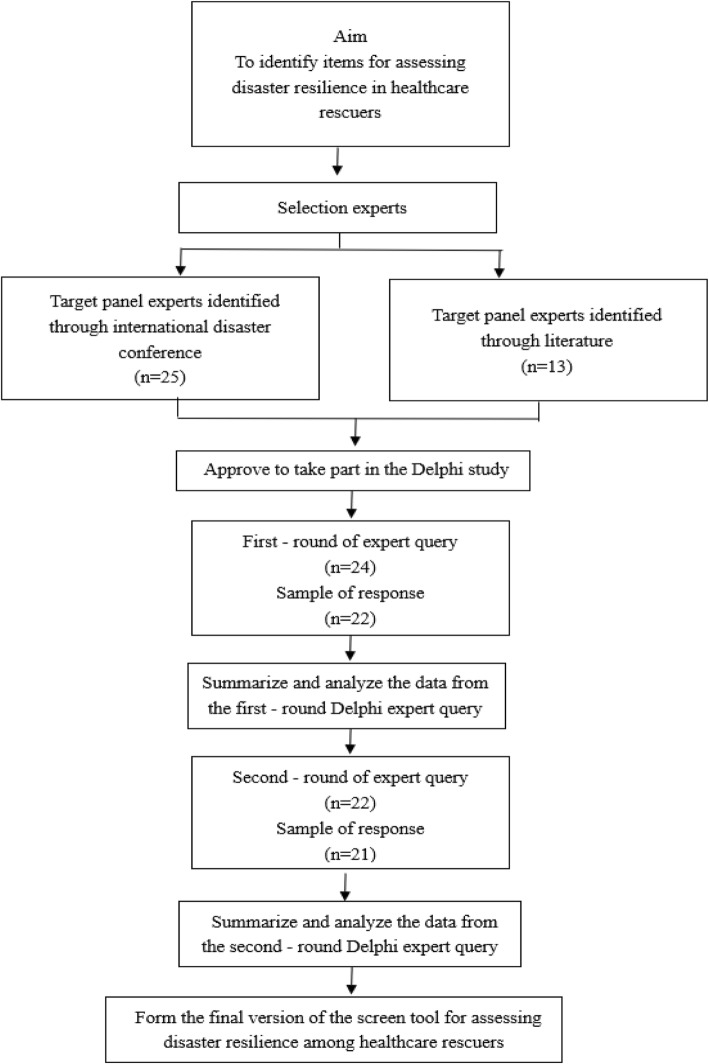
Flow diagram of panel selection and Delphi process

This Delphi survey took place from 4th February to 20th April 2019. A consensus on the items was achieved after two rounds of the survey. A flow diagram of the Delphi process is given in Fig. 1.

Before commencing the first round of the Delphi survey, a total of 38 experts were invited to take part in this modified Delphi study. Twenty-eight of them responded, of whom 24 had the willingness and time to become involved in this study. Emails were then sent to these 24 experts. A total of 22 of them gave feedback on the prototype tool, although a few required email reminders before doing so (for a response rate of 91.7%). In the second round, emails were sent to these 22 experts. In the end, a total of 21 experts completed the Delphi survey (for a response rate of 95.5%). The demographic characteristics of the panel experts are presented in Table 1.

**Table 1:** The characteristics of the experts in panel

Characteristics	Demographics	First-round (*n* = 22)	Second-round (*n* = 21)
		n (%)	n (%)
Gender	Male	3 (13.6)	3 (14.3)
	Female	19 (86.4)	18 (85.7)
Age (years)	31–40	3 (13.6)	3 (14.4)
	41–50	11 (50)	10 (47.6)
	51–60	2 (9.1)	2 (9.5)
	Over 60	4 (18.2)	4 (19.0)
	No response	2 (9.1)	2 (9.5)
Nationality	National (China mainland, Hong Kong, Taiwan)	16 (72.7)	16 (76.2)
	International (America, Japan, South Korea, Australia)	6 (27.3)	5 (23.8)
Specialty	University academic	11 (50.0)	10 (47.6)
	Physician	3 (13.6)	3 (14.4)
	Nurse	7 (31.8)	7 (33.2)
	Disaster management	1 (4.6)	1 (4.8)
Title of the job	Professor	10 (45.4)	9 (42.9)
	Associate professor	8 (36.4)	8 (38.1)
	Lecturer	4 (18.2)	4 (19.0)
Education level	Bachelor	2 (9.1)	2 (9.5)
	Master	5 (22.7)	5 (23.8)
	Doctoral/ PhD	14 (63.6)	13 (61.9)
	Post-doctoral	1 (4.6)	1 (4.8)
Work area	Disaster nursing	10 (45.4)	9 (42.9)
	Disaster medicine	1 (4.6)	1 (4.8)
	Disaster management	3 (13.6)	3 (14.3)
	Disaster education	13 (59.1)	12 (57.1)
	Disaster related research	9 (40.9)	7 (33.3)
Disaster working experience (years)	5–10	5 (22.7)	4 (19.0)
	11–15	10 (45.4)	10 (47.6)
	16–20	1 (4.6)	1 (4.8)
	over 20	4 (18.2)	4 (19.0)
	No response	2 (9.1)	2 (9.5)

Table 2 shows the median, interquartile range, and level of agreement of all items in the prototype tool in the first round of the Delphi study. Regarding the 66 items in the first draft of the prototype tool, a total of 81 comments were received from experts. The researchers held a meeting to discuss these comments. As a result of the discussion, a total of 17 items were accepted and their wording was revised as suggested, 25 items were merged into 11 items, and 3 items were added. Another 19 items were regarded as irrelevant by 8 experts or did not meet the criteria for a consensus and were deleted as suggested. The Kendall’s coefficient of concordance (W) of the first round of the Delphi survey was calculated to be 0.21 (*P* < 0.01). The prototype tool was reduced from 66 to 36 items after the first round of the survey.

**Table 2:** The median, interquartile range, and the level of agreement of items in the first-round query

Domains	Items	Median	Interquartile range	Level of agreement
Optimism	1. I often think that difficulties are everywhere during and after rescue work (R).	4.00	1.00	0.81
	2. I can’t change the reality (R).	4.00	2.00*	0.62*
	3. I tend to think things in a positive way when I witness bad happenings at disaster site.	4.00	1.00	0.95
	4. I tend to think that there will be solutions to all the encountered problems during and after deployment.	4.00	1.00	0.95
	5. I have a bright outlook for the future.	4.00	1.00	1.00
Altruism	6. I had a desire to help others.	4.00	1.00	0.95
	7. I have a strong will to offer help to others after disaster occurred.	4.00	0.00	1.00
	8. I am inspired to work in disaster areas.	4.00	0.00	1.00
	9. I am very willing to offer help to victims of the disaster.	4.00	0.00	0.95
	10. I am honored to work in the frontline to offer my help to my people.	4.00	0.00	1.00
	11. I will devote myself to the disaster rescue work.	4.00	0.00	0.95
	12. I feel that it is a personal responsibility to help others after disaster	4.00	1.00	0.90
Preparations for disaster	13. I have insurance covered during deployment.	4.00	1.00	0.86
	14. I was offered psychological training before a rescue mission.	4.00	0.50	0.90
	15. I received disaster-related knowledge and skills such as Psychological First Aid (PFA) and field survival skills.	4.00	0.00	1.00
	16. I read from books on positive psychology in order to remain calm when facing difficulties.	3.00*	1.00	0.90
	17. I learned the coping strategies and stress release approach before deployment.	4.00	1.00	1.00
	18. I usually do exercise to keep healthy.	3.00*	1.50*	0.76
Social support	19. My relatives provide help for me during my deployment.	4.00	1.00	0.86
	20. My family give me strong supports.	4.00	1.00	0.95
	21. My family will share the joy with me when I came back from disaster site.	4.00	1.00	0.81
	22. My friends accompany me to overcome the challenges.	3.00*	1.50*	0.76
	23. I have some close friends/family members encouraging me.	3.00*	1.00	0.90
	24. During the rescue phrase, my colleagues and I help each other.	4.00	0.75	0.95
	25. My hospital takes care of my family during my deployment.	4.00	1.00	0.81
	26. When I have problems, I always feel that I have no one to count on (R).	3.00*	2.75*	0.62*
	27. I can ask for help when something bad happens.	4.00	0.75	0.95
	28. The local authority has provided us with food and medical equipment.	4.00	2.00*	0.67*
	29. I feel isolated from my colleagues and rescue team members (R).	3.00*	3.00*	0.48*
Perceived control	30. How things go in my life depends on my own actions.	4.00	1.00	0.86
	31. My decision makes a real difference in how things turn out in the end.	4.00	0.50	0.95
	32. My own future depends on myself.	4.00	1.00	0.81
	33. I can achieve my goal by working hard.	4.00	1.00	1.00
	34. I can control the results by preparing for disaster rescue work.	4.00	1.00	0.95
	35. More people can be saved when I work seriously and thoroughly in the disaster site.	4.00	1.00	0.90
	36. No more survivor can be saved no matter how hard I work (R).	3.00*	2.00*	0.62*
Self-efficacy	37. I felt empowered and have realized my clinical experience became very useful whilst participating in rescue work.	4.00	0.50	1.00
	38. I can easily adjust in a difficult situation in the rescue site.	4.00	1.00	1.00
	39. I can cope well with unexpected problem in the process of disaster rescue.	4.00	1.00	1.00
	40. I believed in myself during carry out the rescue activity.	4.00	1.00	0.95
	41. In the rescue site, my self-esteem was enhanced by communicating with colleagues about the situation of my family.	3.00*	1.00	0.76
	42. I become psychologically stronger within the rescue team.	4.00	1.00	0.90
	43. I know that I will bounce back no matter how the difficult is.	4.00	1.00	0.90
	44. I am a person who can make the right decisions in most cases.	4.00	1.00	0.95
	45. I am the excellent rescue workers.	4.00	1.00	0.90
	46. I make mistake often during disaster rescue. (R)	3.00*	3.00*	0.62*
Coping strategies	47. I always try to find a way to do what’s necessary to carry on.	4.00	1.00	1
	48. I often look for creative solutions in the face of disastrous events.	4.00	0.00	0.95
	49. I always give up in the face of difficulties (R).	3.00*	2.50*	0.67*
	50. When I failed to address problems, I think of other ways to address.	4.00	1.00	0.90
	51. If disaster occurs, I will find a place to hide and prevent myself from injuries.	3.00*	1.75*	0.71
	52. When life is threatened, I will remain calm and would actively face the problem.	4.00	0.75	0.95
	53. Whenever I encountered difficulties, I would remind myself there is always a solution to the problem.	4.00	0.75	0.90
	54. As time goes by, I will slowly forget about the disaster.	3.00*	1.5*	0.76
	55. I have to wait calmly for the rescue team if I was trapped.	3.00*	1.00	0.86
	56. I would adjust my own thoughts to remain a calm attitude in disaster rescue.	4.00	1.00	1.00
	57. When I am tired from work, I would have a cigarette or go fishing which I won’t think of anything.	4.00	1.00	1.00
Positive growth	58. I have growth from the rescue work	3.00*	2.50*	0.48*
	59. I tend to see recue work as a challenge.	4.00	1.00	0.90
	60. I find meaning from my deployment.	4.00	0.50	1.00
	61. I find strength from disaster recue work.	4.00	1.00	0.95
	62. I tend to think Living in the moment is important.	3.00*	2.00*	0.71
	63. After returning from disaster deployment, I have a more harmonious family life.	4.00	1.00	0.86
	64. My colleagues and I are like buddies after deployment.	4.00	1.00	0.90
	65. I cannot be afraid of disaster and just have to accept whatever life brings	3.00*	2.00*	0.67*
	66. I think that having a religious belief would be helpful in disastrous situations.	3.00*	2.00*	0.62*

Note: *R* reversed description; * = not meet the criteria of consensus

Table 3 shows the median, interquartile range, and level of agreement of all items in the disaster resilience tool following the second round. Another meeting was held among the researchers to discuss the comments and suggestions that were received. A total of 8 items were deleted since a consensus was not reached on them. A total of 12 items were accepted but the wording of these items was changed to achieve more precision in proper English, 4 items were merged into 2 items due to overlap, and 1 item was added after the discussion and approval from all researchers. The Kendall’s coefficient of concordance (W) in the second round of queries was 0.33 (*P* < 0.01). The final version of a 27-item tool for measuring the disaster resilience of healthcare rescue workers was established (Table 4).

**Table 3:** The median, interquartile range, and the level of agreement of items in the second-round query

Domains	Items	Median	Interquartile range	Level of agreement
Optimism	1. I think that difficulties are everywhere during and after rescue work **(R)**.	4.00	1.00	1.00
	2. I tend to think that problems confronted before, during, and after deployment will be addressed.	4.00	1.00	1.00
	3. I know that I will bounce back and get better no matter how the difficult the situation is, with helps from others.	4.00	1.00	1.00
	4. I have a bright outlook for the future.	3.00*	2.00*	0.65*
Altruism	5. I have a desire to help the victims /survivors after disaster occurred.	4.00	0.75	1.00
	6. I am honored to work in the frontline to offer my help to those who are affected by disaster.	4.00	0.75	1.00
	7. My chance of being promoted should be increased if I offer to work in the disaster areas (**R**).	3.00*	2.00*	0.65*
	8. I feel that it is a personal responsibility to help others after disaster.	3.50	1.00	0.95
	9. More people can be saved when I work with all my heart at the disaster site.	3.00*	1.75*	0.75*
Preparations for disaster rescue	10. I (will) have special personnel life accident and organization liability insurance covered during deployment.	3.00*	2.75*	0.65*
	11. I am certain of my family’s safety while I am deployed.	3.50	1.00	0.90
	12. I have a good command of knowledge and skills for disaster rescue such as medical rescue skills, Psychological First Aid (PFA), and field survival skills.	4.00	0.00	1.00
	13. I have no idea how a tent/field hospital is constructed (R)	3.00*	3.00*	0.55*
	14. I feel unprepared physically for disaster rescue (R).	4.00	1.00	0.95
Social support	15. My family (will) provide me with strong support during and after mission.	4.00	0.75	1.00
	16. Co-workers (will) help me overcome the challenges in the disaster site.	4.00	0.75	1.00
	17. I have some close friends who will provide me with much encouragement.	4.00	1.00	0.95
	18. My work unit (will) provide support with my family when I work in disaster site.	4.00	1.00	1.00
	19. I have no one to count on if something bad would happen to me(R).	3.00*	2.75*	0.70*
Perceived control	20. How things (will) go during and after deployment depends on my own actions.	3.50	1.00	0.85
	21. I can handle the situation at the disaster site.	4.00	1.00	1.00
	22. I can remain a calm attitude during a disaster rescue.	4.00	1.00	1.00
Self-efficacy	23. I feel confident that my clinical skills (will be) are of good use for disaster work.	4.00	0.00	1.00
	24. I can cope well with unexpected problems during a disaster rescue.	4.00	1.00	1.00
	25. I believe in my capability during the rescue activity.	4.00	1.00	1.00
	26. I can keep calm at the disaster site.	4.00	1.00	0.95
	27. I am an excellent rescue worker.	4.00	1.00	1.00
Coping strategies	28. I always try to find a way to address the problems that I am confronted with for the duration of the disaster events.	4.00	0.75	0.95
	29. When life is threatened, I lose my temper and blame others (**R**).	4.00	1.00	0.85
	30. when I feel depressed during and after deployment, I (will) always express my feelings to friends.	3.50	1.00	0.85
	31. When experiencing anxiety during or after a disaster, I (will) try to do something such as watch a movie/go fishing/have a cigarette to keep my mind occupied.	3.00*	2.00*	0.65*
Positive growth	32. I have gained insight about life from the rescue work.	4.00	0.00	1.00
	33. I tend to see recue work as a challenge after deployment.	4.00	1.00	1.00
	34. I find meaning from my deployment.	4.00	0.00	1.00
	35. After returning from disaster deployment, I have a more harmonious family life.	4.00	1.00	1.00
	36. My colleagues and I are like buddies after deployment.	3.00*	2.00*	0.70*

Note: *R* reversed description; * = not meet the criteria of consensus

**Table 4:** The final version of the disaster resilience tool for healthcare rescuers after a two-round Delphi survey

Domains	Items
Optimism	1. I think that difficulties are everywhere during and after rescue work. **(R)** 2. I tend to think that problems confronted before, during, and after deployment will be solved. 3. I know that I will bounce back and get better no matter how the difficult the situation is, with help from others.
Altruism	4. I have a desire to help the victims / survivors after a disaster has occurred. 5. I am honored to work in the frontline to offer my help to those who are affected by disaster. 6. I feel that it is a personal responsibility to help others after disasters.
Preparations for disaster rescue	7. I am certain of my safety and that of my family while I am deployed. 8. I have sufficient knowledge to assess disaster risks, and have disaster rescue skills such as medical rescue skills, knowledge of Psychological First Aid (PFA), ethical rules, and field survival skills. 9. I am emotionally well-prepared for disaster rescue. 10. I feel physically unprepared for the disaster relief. (**R**)
Social support	11. My family will provide me with strong support during and after my disaster relief work. 12. Co-workers will help me to overcome challenges in the disaster site. 13. I have some close friends who will provide me with much encouragement. 14. My work unit will provide support to my family, and to me if necessary, when I work in disaster sites.
Perceived control	15. How things go during and after deployment will depend on my own actions. 16. I can handle various situations at a disaster site. 17. I can remain calm during a disaster rescue.
Self-efficacy	18. I feel confident that my clinical skills (will be) are of good use for disaster work. 19. I can cope well with unexpected problems during disaster rescues. 20. I am a competent rescue worker.
Coping strategies	21. I always try to find ways to address problems during disaster events. 22. When a victim’s / survivor’s life is threatened, I lose my temper and blame others. (**R**) 23. I am willing to express my emotions to others if I am upset.
Positive growth	24. I have gained insight about life from the rescue work. 25. I tend to see rescue work as a challenge after deployment. 26. I find meaning from my deployment. 27. After returning from deployment to a disaster site, I have a more harmonious family life.

Note: *R* reversed description

After two rounds of the modified Delphi survey, the I-CVI for the disaster resilience tool for healthcare rescuers ranged from 0.85 to 1.0. The S-CVI/UA and S-CVI /Ave were 0.69 and 0.97, respectively. The consensus of all items for the disaster resilience tool was reached. Therefore, the Delphi experts survey was completed after two rounds.

## Discussion

To the best of our knowledge, this is the first study to validate a tool for measuring the disaster resilience of healthcare rescuers through the use of a Delphi survey to gauge the views of experts in the field of disaster work and research. After two rounds of a web-based modified Delphi survey, a 27-item tool for screening the disaster resilience of rescuers was identified (Table 4). The outcome of this study, the measuring tool, can be used as a reference to recruit and identify disaster rescue workers who have the characteristics of disaster resilience, or as a tool to evaluate the effectiveness of resilience training programs for healthcare disaster rescue workers [49]. The modified approach adopted in this Delphi survey is considered superior to the original approach because it is highly effective and less time consuming [50, 51].

Having an “expert panel” is central to the process of the Delphi technique, although there are no standard criteria for determining expertise [52]. In the current study, the panel of experts comprised people from seven countries/cities who are in diverse professions, such as university academics, physicians, and nurses. They are from the fields of disaster nursing, disaster medicine, disaster education, disaster management, and disaster research. A panel consisting of experts from different geographical locations [25] and areas of professional expertise [27, 53] will produce better results than a panel comprised of those from the same field [54].

Among the experts in our panel, some had taken part in national and / or international disaster rescue work, and the panel as a whole reflected the full range of stakeholders with a common interest [55]. These experts can also be regarded as “consumers” with lived experience of disaster rescue [23]. Therefore, the measuring tool from this Delphi study can be applied in all countries by disaster practitioners, educators, researchers, and management personnel.

Through their active and very timely responses, the expert panel in this study showed strong motivation and interest in taking part in the Delphi survey. Although there is no strict rule for what is considered an acceptable response rate for Delphi studies, a response rate of 70% is suggested necessary for each round [19]. The response rates of the two rounds of Delphi surveys in this study were higher than 90%, and a great number of constructive suggestions and comments were received, indicating the experts’ considerable enthusiasm and interest in this topic. This may be related to the fact that most of the experts also considered it is important to have such measuring tool, and that they were approached in disaster-related international conferences and invited in person. The short timeframe between the two rounds of surveys (2 to 3 weeks) also served to keep the subject fresh in their minds and prevent fatigue. This also helped to enhance the content validity of this modified Delphi study, ultimately strengthening the validity of the results [24].

The Content Validity of this study was good. The I-CVI of the included items varied from 0.85 to 1.00, which is higher than the recommended level of 0.78, suggesting that the content of each item of the disaster resilience tool is excellent [48]. The S-CVI/UA was 0.69, which did not reach the acceptable value of 0.8. This can be explained by the larger sample size of experts in this study for the calculation of S-CVI/AV, and the consequent risk of disagreements [48]. The S-CVI /Ave was as high as 0.97, indicating that the content validity of the whole scale is excellent. Thus, the content validity of this disaster resilience tool can be regarded as excellent, although some of the items may need to be slightly revised based on the comments or suggestions of the expert panel.

After two rounds of surveys, the experts reached a consensus on all of the items that were finally included in the disaster resilience tool. The median range of all of the included items was 3.50 to 4.00 and the interquartile range was 0.00 to 1.00, while the statement of all items demonstrated ≥85% agreement, indicating good consensus [41–43]. The Kendall coordination coefficient W, used to assess the agreement among several expert evaluators [56], was 0.207 in the first round and 0.33 in the second round, with *P* < 0.01. This suggests a highly significant level of consensus among the experts in the panel.

As with any research, there are some limitations to this study that need to be acknowledged. First, to prevent peer influence in a Delphi study, each member of the panel of experts should not know about the others [57]. However, the members of the expert panel in this study were mainly approached during international conferences. Thus, it was inevitable that some of these experts would know about each other, which means that absolute anonymity could not be achieved in this study. Nevertheless, all of the experts rated the items of this tool independently at their own location, so the results of the rating of each item were anonymous. Second, because the experts lived in different countries/cities, no face-to-face meetings were held among them in the process of carrying out this modified technique. Finally, the opinion of the panel of experts in this study may not be representative of all experts within the field of disaster studies, as experts from other countries that are frequently affected by disasters, such as India and Indonesia, were not involved in this study.

In spite of its limitations, this study provides significant information for disaster management. In the next step, this developed disaster resilience tool is to be validated for its psychometric quality, including its reliability and validity, in a cross-sectional study on healthcare disaster rescue workers. Researchers, management, and policy makers will then have a validated tool to use in recruiting or assessing disaster resilience among healthcare rescuers.

The validation process would include the following: translating the tool into languages other than English if it is to be validated in countries where English is not the main language, conducting a pilot test among a sample of disaster healthcare rescuers to assess the clarity and pertinence of the items in the language of the country where the tool is being tested and, finally, conducting a cross-sectional survey among a large sample of disaster healthcare rescuers to test the reliability and construct validity of the tool.

## Conclusion

This study has established a tool for assessing the disaster resilience of healthcare rescuers, using a modified Delphi technique. The tool is a scale made up of a total of eight domains and 27 items. The panel of experts reached a consensus on all of the items in this scale, and the items and the overall scale were found to have excellent content validity. A study to establish the psychometric properties of this scale is needed in the next step before it can be used as a tool in the recruitment and management of disaster healthcare rescue workers.

## Data Availability

The data were hold by the first author, if it is needed, please contact at xiaorong.mao@connect.polyu.hk.

## Abbreviations

APEDNN: Asia Pacific Emergency and Disaster Nursing Network
CD-RISC: Connor-Davidson Resilience Scale
CVI: Content validity index
PTSD: Posttraumatic stress disorder
SFDRR: Sendai Framework for Disaster Risk Reduction
SPSS: Statistical Package for the Social Scientists
USA: United States of America.
WSDN: World Society of Disaster Nursing
